# An artificial neural network approach to detect presence and severity of Parkinson’s disease via gait parameters

**DOI:** 10.1371/journal.pone.0244396

**Published:** 2021-02-19

**Authors:** Tiwana Varrecchia, Stefano Filippo Castiglia, Alberto Ranavolo, Carmela Conte, Antonella Tatarelli, Gianluca Coppola, Cherubino Di Lorenzo, Francesco Draicchio, Francesco Pierelli, Mariano Serrao

**Affiliations:** 1 Department of Occupational and Environmental Medicine, Epidemiology and Hygiene, INAIL, Monte Porzio Catone Rome, Rome, Italy; 2 Department of Medico-Surgical Sciences and Biotechnologies, University of Rome Sapienza, Latina, Italy; 3 IRCSS Fondazione Don Carlo Gnocchi, Milan, Italy; 4 Department of Human Neurosciences, University of Rome Sapienza, Rome, Italy; Emory University, UNITED STATES

## Abstract

**Introduction:**

Gait deficits are debilitating in people with Parkinson’s disease (PwPD), which inevitably deteriorate over time. Gait analysis is a valuable method to assess disease-specific gait patterns and their relationship with the clinical features and progression of the disease.

**Objectives:**

Our study aimed to i) develop an automated diagnostic algorithm based on machine-learning techniques (artificial neural networks [ANNs]) to classify the gait deficits of PwPD according to disease progression in the Hoehn and Yahr (H-Y) staging system, and ii) identify a minimum set of gait classifiers.

**Methods:**

We evaluated 76 PwPD (H-Y stage 1–4) and 67 healthy controls (HCs) by computerized gait analysis. We computed the time-distance parameters and the ranges of angular motion (RoMs) of the hip, knee, ankle, trunk, and pelvis. Principal component analysis was used to define a subset of features including all gait variables. An ANN approach was used to identify gait deficits according to the H-Y stage.

**Results:**

We identified a combination of a small number of features that distinguished PwPDs from HCs (one combination of two features: knee and trunk rotation RoMs) and identified the gait patterns between different H-Y stages (two combinations of four features: walking speed and hip, knee, and ankle RoMs; walking speed and hip, knee, and trunk rotation RoMs).

**Conclusion:**

The ANN approach enabled automated diagnosis of gait deficits in several symptomatic stages of Parkinson’s disease. These results will inspire future studies to test the utility of gait classifiers for the evaluation of treatments that could modify disease progression.

## 1. Introduction

Parkinson’s disease (PD) is a progressive neurodegenerative disorder characterized by a broad spectrum of motor and non-motor features [1, 2]. Gait deficits represent one of the most debilitating aspects in people with Parkinson’ s disease (PwPD); these inevitably decline over the course of the disease, strongly increase the risk of falls, and greatly reduce patient autonomy and quality of life [3–5]. The mechanism underlying gait impairment is complex and multifactorial and is caused by multi-system lesions involving both the dopaminergic and non-dopaminergic mechanisms related to bradykinesia, rigidity, impaired balance and postural control, visual motor deficiency, and cognition [6, 7]. Given the importance of autonomous and effective locomotion in humans [8] and considering the socio-economic burden of gait deficits [9], clinician should strive to optimize pharmacological treatments and rehabilitation interventions to improve gait function.

In recent years, gait analysis has become an essential tool for objective evaluation of gait changes induced by pharmacological and rehabilitative interventions [10]. Furthermore, it is a valuable technique to classify gait patterns according to a specific disease or group of diseases [3, 11–13] or the disease severity [14], allowing more accurate assessment of the quantitative gait measures to the qualitative clinical features for clinical practice purposes. An innovative approach to address this issue is the use of quantitative machine-learning techniques such as artificial neural networks (ANNs), which are mathematical models that represent a distributed adaptive system built using multiple interconnecting processing elements, just as real neural networks do [15, 16]. In this model, the processing elements (neurons) are distributed in several layers: each neuron receives signals processed and transmitted by neurons in the preceding layer and, in turn, processes and transmits them to the next layer [15, 16].

ANNs have been used in many research fields such as psychology, robotics, biology, computer science, and ergonomics [17, 18] and, more recently, as diagnostic tools in several clinical conditions, including colon or colorectal cancer [19, 20], multiple sclerosis [21, 22], pancreatic disease [23], gynecological diseases [24], and early diabetes [25]. However, few studies have attempted to identify and classify gait deficits using machine-learning approaches in neurological disorders, including Huntington disease [26] and PD [27–29]. Particularly, with regard to PwPD, most of the published studies investigated two-group gait pattern classifications, differentiating PwPD from healthy subjects [28, 30, 31], or performed multiclass classification according to the disease severity using the Unified Parkinson’s Disease Rating Scale [32, 33]. However, none of these previous studies specifically searched for those gait parameter features able to categorize the gait pattern according to disease progression using the Hoehn and Yahr (H-Y) staging system [34].

The H-Y scale has several strengths and is considered the reference standard for disability and impairment measurements [35]. It is significantly correlated with dopaminergic loss [35, 36], cerebral spinal fluid and serum alpha-synuclein levels [37], quality of life measurements [38], and motor performance [39]. Changes in the H-Y stage carry prognostic significance and influence clinician-based interventions [40]. For instance, the European Guidelines on PD rehabilitation suggest differentiating the rehabilitation program according to the disease progression; for instance, promoting balance and gait training for H-Y stages 2 to 4 [41]. Therefore, identifying the specific gait pattern for each disease stage may allow monitoring of the changes induced by pharmacological and rehabilitation treatment in each stage, taking into account only those meaningful parameters able to classify gait deficit in PwPD. When studying gait features, it is classically adopted a univariate approach, whereby measurement outcomes are considered independently. This data redundancy may be highly time–consuming and increase the risk of losing important information [42]. In this view, machine learning approaches may overcome this limitation, allowing to explore the optimal combination of gait characteristics reducing the computational demand at the same time [42, 43].

Our study aimed to i) develop a diagnostic algorithm based on machine-learning techniques (i.e., ANNs) able to classify the gait deficit of PwPD according to the disease progression as evaluated by the H-Y staging system and ii) identify the minimum set of gait time-distance and kinematic parameters able to distinguish the H–Y stage gait pattern from each other.

## 2. Materials and methods

### 2.1 Patients

This study enrolled 76 PwPD (age: 69.68±8.92 years). Clinical evaluation of the severity of Parkinsonism included neurological and functional assessments using the UPDRS III (18±12) and the H-Y staging system (20 with H-Y = 1 (age: 66.3±9.3 years; height: 163.8±8.0 cm; weight: 74.3±15.2 kg), 17 with H-Y = 2 (age: 72.9±8.4 years; height: 164.4±9.6 cm; weight: 72.9±12.7 kg), 27 with H-Y = 3 (age: 68.9±8.9 years; height: 162.9±8.7 cm; weight: 75.0±11.4 kg), and 12 with H-Y = 4 (age: 73.8±7.2 years; height: 162.3±7.9 cm; weight: 71.1±10.8 kg)). The inclusion criteria were: a diagnosis of idiopathic PD according to the UK Brain Bank Diagnostic Criteria [44], H-Y stages 1–4, stable drug program (taking current medication for at least 2 weeks) and the ability to walk independently on at least the laboratory pathway without showing freezing of gait. The exclusion criteria were: cognitive deficit (defined as scores <24 on the Mini-Mental State Examination, moderate or severe depression (defined as scores ≥20 on the Beck Depression Inventory, and presence of orthopedic and/or other gait-influencing conditions such as arthrosis or total hip joint replacement. Medication was kept constant throughout the trial period and all interventions were performed at the same time of day for each patient during the “ON phase.” The participants were asked to maintain their usual activity levels and current medication dosage when not in the laboratory. The assessments for both clinical and instrumental evaluations were not involved in the treatment of the patients and were blinded to the time of the evaluation. The patients were on oral levodopa (16 patients), dopamine agonists (27 patients), or both (33 patients) and were recorded to be in the “ON phase.” The levodopa equivalent dose was 571.92 ± 317.2 mg [45].

Sixty-seven healthy subjects (HS) were enrolled as the healthy control group (age, 69.84±8.79 years).

All participants provided written informed consent before taking part in the study, which was approved by a local ethics committee (Sapienza University of Rome, Policlinico Umberto I, UP 00988_2020) and complied with the principles of the Declaration of Helsinki.

### 2.2 Gait analysis

An optoelectronic motion analysis system (SMART-DX 6000 System, BTS, Milan, Italy) consisting of six infrared cameras (sample frequency, 340 Hz) was used to detect the movement of twenty-two passive spherical markers covered with reflective aluminum powder (15 mm in diameter) placed over prominent bony landmarks, according to the International Society of Biomechanics recommendations [46] and Davis’s protocol [47]. Using double-adhesive tape, the markers were placed over the cutaneous projections of the spinous processes of the seventh cervical vertebra and sacrum and bilaterally over the acromion, anterior superior iliac spine, great trochanter, lateral femoral condyle, fibula head, lateral malleolus, and metatarsal head. In addition to markers directly applied to the skin, sticks or wands, varying in length from 7 to 10 cm, placed at 1/3 of the length of the body segment (femur and leg) were also used [47].

### 2.3 Experimental procedure

The patients and controls were asked to walk barefoot at a comfortable, self-selected speed along a walkway approximately 10 m in length while looking forward. We provided only general and qualitative instructions because we were interested in natural locomotion. Before the recording session, the subjects practiced for a few minutes to familiarize themselves with the procedure. We instructed the HCs to also walk at low speeds to compare the parameters between groups without potential velocity bias [48]. At least ten trials were recorded for PwPD. At least ten trials at a self-selected speed and ten trials at a slow speed were recorded for HCs. Groups of three trials were separated by 1-min rest periods to avoid fatigue. To avoid potential velocity bias, gait speed was matched between groups as follows: for each healthy subject, we considered only those trials in which gait speed fell within the range identified by PwPD mean gait speed ± standard deviation.

### 2.4 Data analysis

After each acquisition performed by Smart Capture (BTS, Milan, Italy), three-dimensional marker trajectories were reconstructed using a frame-by-frame tracking system (SMART Tracker, BTS, Milan, Italy). Then, the data were processed using SMART Analyzer (BTS, Milan, Italy) and MATLAB (version 7.10.0, MathWorks, Natick, MA, USA) software.

In this study, heel strike and toe-off events were determined by maximum and minimum limb angle excursions. The limb angle was calculated as the angle between the vertical axis from the greater trochanter and a vector drawn from the greater trochanter to the lateral malleolus projected on the sagittal plane: a 0° limb angle meant that the leg was positioned vertically under the body; positive angles denoted flexion (i.e., limb positioned in front of the vertical axis) and negative angles denoted extension (i.e., limb positioned behind the vertical axis) [49]. After this preprocessing procedure, the time-distance and kinematic parameters were evaluated and the kinematic data were normalized to the duration of the gait cycle (defined as the interval between two successive foot contacts of the same leg) and interpolated to 101 samples using a polynomial procedure.

#### 2.4.1 Time-distance parameters

The following time-distance gait parameters were calculated for each subject and for each stride: walking speed (m/s), cadence (step/s), step width (m), step length (m) (defined as the distance from the heel strike of a limb and the subsequent heel strike of the other limb), stance, swing, and double support phase durations (expressed as percentages of the gait cycle duration). Step length and step width were normalized to the limb length of each subject. For each subject, the average value of each gait feature was calculated.

#### 2.4.2 Kinematic data

The anatomical joint angles of the hip, knee, ankle, trunk, and pelvis (frontal, sagittal, and transverse planes) and the corresponding ranges of motion (RoMs) of the joints (defined as the differences between the maximum and minimum values during the gait cycle) were computed. For each subject, the average value of each RoM was calculated.

#### 2.4.3 ANN approach for the diagnosis and staging of the gait deficit in PwPD

*HS vs PwPD classification*. A principal component analysis (PCA), using a threshold of 98% on the cumulative variance was used to define a subset of features starting from all time-distance and kinematic HS and PwPD features [50].

Then, an artificial neural network (ANN) approach was used for diagnosis of Parkinson disease using the features selected by PCA.

We trained different topologies of feedforward networks with different numbers of hidden layers (HL) and different numbers of neurons (N) in each HL. The number of HL varied in the range of 1–3, while the number N in each HL varied based on the number of N in the first hidden layer (N_L1_), N was set to two different values (20 and 50, respectively), and the number of nodes in the other HL (when defined) was 1/2 and 1/3 of N for the second (N_L2_) and third (N_L3_) HLs, respectively. Thus, the combination of L layers and N nodes in the first HL hidden layer led to the six different network architectures. The output set consisted of an orthogonal coding of the two values (HS vs PwPD): OUT1 = [1 0] and OUT2 = [0 1] (one-hot classification coding scheme).

Networks were trained with a supervised approach using the Levenberg-Marquardt back-propagation algorithm, stopping when at least one of the following conditions was met: 1000 iterations, 10^−6^ mean square error, or six consecutive fails on the validation set [17]. To verify the repeatability of our results, each of the six network topologies was trained ten times by using a random 10% of samples as the validation set and a random 10% as the testing set. For each trained network, a confusion matrix was calculated based on the real value (HS or PwPD) and the one estimated on the randomly extracted testing set.

**Fig 1:**
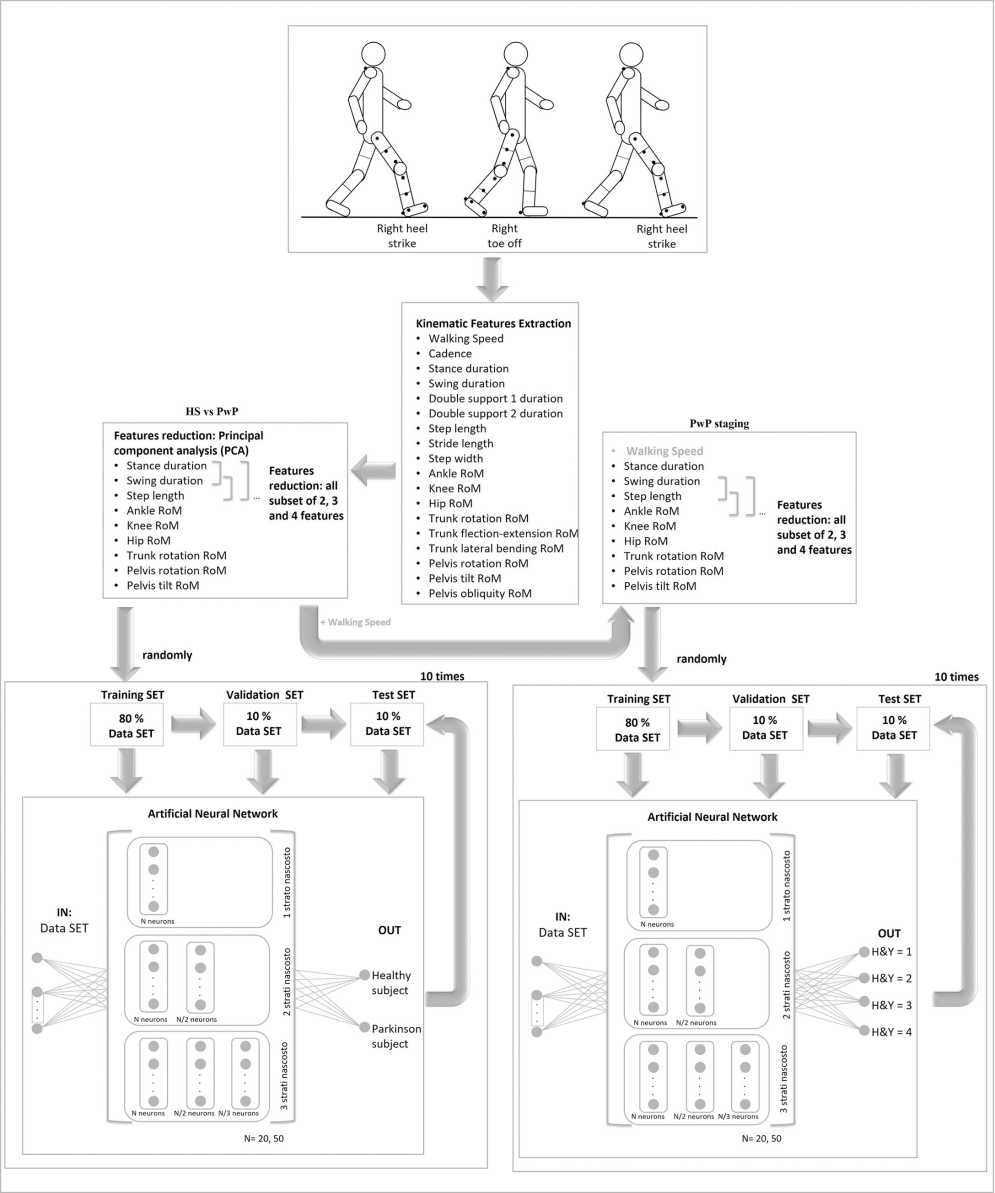
Description of experimental set-up and methodological approach. A schematic description of the walking and artificial neural network method used to map time-distance and kinematic features on the H&Y (1, 2, 3 and 4) levels.

The mean 2×2 confusion matrix was then obtained by averaging the confusion matrixes of the trained ANNs. A performance parameter (P) was calculated as the mean (%) of the elements on the diagonal of the mean confusion matrix, where 100% indicates the absence of misclassifications [17]. Furthermore, the sensitivity and specificity of each group were calculated. The entire system is schematically described in Fig 1.

Then, to reduce the features, we also used subsets of features from the selected features with PCA and, for each subset, we trained the six ANNs ten times to evaluate the confusion matrix and performance. We started with subsets of all combination of two features and continued until we identified a subset whose performance was no different from that of the set with all features selected with PCA (Fig 1).

*PD staging classification*. An ANN approach was also used to stages the gait deficits in PD in terms of the H-Y scale using the features selected by PCA (2.4.3.2) and walking speed.

We trained the six different topologies of feedforward networks as described in section *2*.*4*.*3*.*1*. The output set consisted of an orthogonal coding of four values of H-Y: OUT1 = [1 0 0 0], OUT2 = [0 1 0 0], OUT3 = [0 0 1 0], and OUT4 = [0 0 0 1] (one-hot classification coding scheme). For each trained network, a confusion matrix was calculated based on the real H-Y value and the one estimated on the randomly extracted testing set. The mean 4×4 confusion matrix was then obtained by averaging the confusion matrices of the trained ANNs and the P was calculated [17].

As described in section *2*.*4*.*3*.*1*, to reduce the features, we also used subsets of features from among the features selected by PCA and, for each subset, we trained the six ANNs ten times, evaluating the confusion matrix and performance. We started with all combination of two features subsets and continued until we found a subset whose performance was no different from that of the set with all features selected by PCA (Fig 1).

### 2.5. Statistical analysis

The Shapiro–Wilk test for normal distribution was preliminarily executed on all gait parameters. Unpaired two-sample t-tests or Mann–Whitney tests (two-tailed) were used for assessment of between-group differences in the time-distance parameters and joint kinematics values. Then, we performed a two-way analysis of variance (ANOVA) test with L and N as factors to determine the possible significant effects on ANN performance, sensitivity and specificity caused by the listed factor. Separate ANOVAs test were performed for performance, sensitivity and specificity. Post-hoc analysis with Bonferroni’s corrections was performed when significant differences were observed in the ANOVA results. P values < 0.05 were considered statistically significant.

Statistical analysis was performed to check if the results of the performance obtained using all features of PCA differed significantly from those obtained considering the subsets of two, three, or four features.

As a confirmative analysis, independent samples t-tests and univariate ANOVA with Bonferroni post-hoc analysis were performed to test the ability of the identified minimum sets of gait parameters to differentiate between PwPD and HS, and PwPD across the H-Y stages, respectively. Receiver operating characteristic (ROC) curves were plotted to assess the discriminative ability of the identified minimum sets of gait parameters in differentiating PwPD from HS and PwPD across the H-Y stages. Area under the curve (AUC), sensitivity and specificity, and positive (LR+) and negative (LR-) likelihood ratios were calculated. The optimal cutoff points (OCP) for the cumulative indices of the combinations of gait parameters included in the identified sets were calculated as the point of the ROC curve where the sum of sensitivity and specificity was highest. Post-test probabilities were inferred by transforming LRs into odds ratios using the Fagan nomogram [51, 52].

## 3. Results

### 3.1 Time-distance and joint kinematics parameters

The values of the time-distance and joint kinematic parameters are reported in Table 1 for both groups. Compared with HCs, PwPD showed significantly lower step length; stride length; hip, knee, and ankle RoMs; trunk flexion-extension; trunk rotation and pelvis rotation values; and higher cadence.

**Table 1:** Kinematic parameters.

	Parameters	PwPD	HS
**Spatio-temporal parameters**	Gait speed (km/h)	2.87±1.07	3.18±0.91
	Stance duration (*% gait cycle*)	65.66±3.49	65.54±3.13
	Swing duration (*% gait cycle*)	34.34±3.49	34.46±3.13
	1st double support *(% gait cycle*)	15.74±3.68	15.40±3.16
	2nd double support *(% gait cycle*)	15.48±3.42	15.50±3.20
	Step length (*% limb length)*	0.68±0.14*	0.77±0.10
	Step width (*% limb length)*	0.30±0.05	0.28±0.05
	Stride length (*% limb length)*	1.19±0.32*	1.43±0.23
	Cadence	0.89±0.22*	0.77±0.15
**Range of motion**	RoM Hip	32.97±11.89*	37.38±5.23
	RoM Knee	52.28±9.39*	60.09±10.62
	RoM Ankle	24.19±7.26*	32.00±12.05
	Trunk lateral bending [°]	3.31±2.05	4.05±1.66
	Trunk flexion-extension [°]	2.84±1.10*	5.99±2.45
	Trunk rotation [°]	8.64±5.05 *	14.36±11.12
	Pelvis obliquity [°]	4.82±3.37	5.77±2.06
	Pelvis rotation [°]	11.20±8.55*	16.48±13.26
	Pelvis tilt [°]	67.78±21.98*	84.25±24.43

Mean ± SD of time-distance and kinetic parameters in patients with Parkinson disease (PwPD) patients and healthy subjects (HS).

*p-value<0.05.

### 3.2 HS vs. PwPD classification

**Fig 2:**
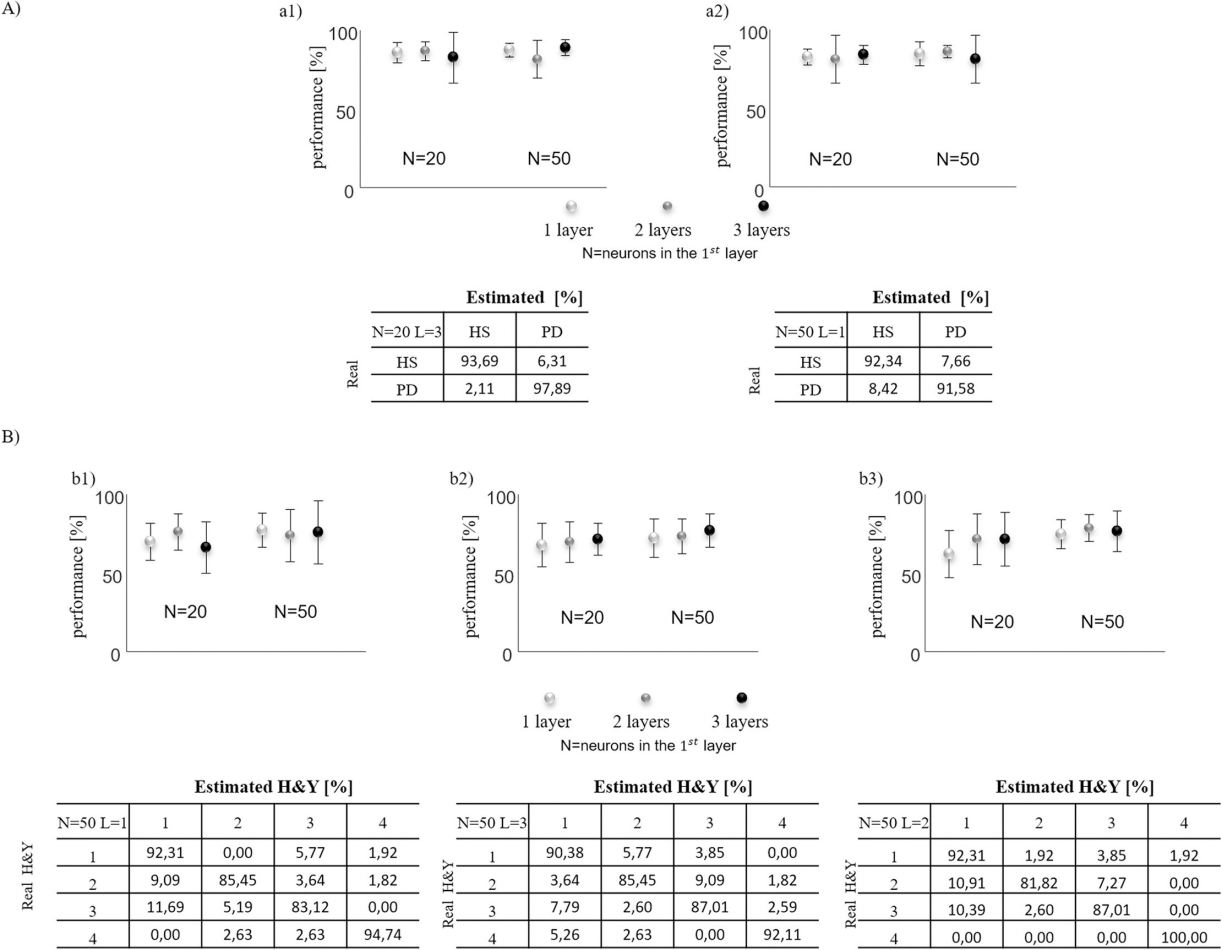
Accuracy of artificial neural networks and the best mean confusion matrix. For diagnosis (A) and staging (B), in the first row the accuracy of artificial neural networks and in the second row the best mean confusion matrixes considering all PCA features as INPUT (a1 and b1) and subset of 2 features (knee RoM and trunk rotation RoM (a2)) and subsets of 4 features (walking speed, hip, knee and ankle RoMs (b2); walking speed, hip, knee and trunk rotation RoMs (b3)). Six different architectures of neural networks were represented by varying the numbers of hidden layers (1, 2, or 3) and the numbers of neurons in each hidden layer based on the numbers of nodes N in the first hidden layer.

The PCA showed that a set of nine features (stance duration, swing duration, step length, ankle, knee, hip, trunk rotation, pelvis rotation, and pelvis tilt RoM) expressed 98% of the cumulative variance of the data (Fig 2A).

The ANNs analysis revealed a mean performance range of 82–98% (Fig 2A), a mean sensitivity range of 81–89% (Table 2), and a mean specificity range of 81–89% (Table 2) for all features detected by PCA for each number of neurons and HL. The number of neurons did not affect the performance (F_1_ = 0.15, p = 0.699), sensitivity (F_1_ = 0.5, p = 0.482), or specificity (F_1_ = 0.5, p = 0.482) in two-way ANOVA. Furthermore, the HL did not affect the performance (F_2_ = 0.48, p = 0.621), sensitivity (F_2_ = 0.32, p = 0.724), or specificity (F_2_ = 0.48, p = 0.621) in two-way ANOVA.

**Table 2:** Sensitivity and specificity.

			HS vs PwPD classification.									PwPD staging classification														
	L	N	HS			PwPD			Mean			H&Y = 1			H&Y = 2			H&Y = 3			H&Y = 4			Mean		
sensibility [%]	1	20	93.2	±	2.03	78.32	±	11.2	85.76	±	5.66	87.88	±	5.52	61.27	±	15.99	69.09	±	8.41	61.05	±	17.4	69.83	±	7.36
	2	20	91.76	±	3.71	81.89	±	8.24	86.83	±	4.77	89.81	±	5.37	66.18	±	15.24	74.29	±	7.08	74.47	±	18.11	76.19	±	8.31
	3	20	91.89	±	4.53	73.58	±	27.59	82.74	±	12.54	87.69	±	9.34	52.36	±	21.66	70.65	±	12.61	53.95	±	21.1	66.16	±	13.1
	1	50	94.19	±	1.48	80.63	±	7.33	87.41	±	3.27	90.77	±	4.23	69.27	±	18.31	75.84	±	7.27	72.89	±	13.19	77.2	±	7.75
	2	50	92.97	±	1.69	70	±	22.21	81.49	±	10.85	86.92	±	11.13	66	±	20.21	71.04	±	12.78	71.58	±	22.32	73.89	±	11.23
	3	50	92.79	±	2.11	84.84	±	8.04	88.82	±	4.21	89.23	±	7.03	67.64	±	24.87	72.34	±	22.87	73.95	±	24.79	75.79	±	16.41
specificity [%]	1	20	78.32	±	11.2	93.2	±	2.03	85.76	±	5.66	72.32	±	7.03	92.12	±	5.05	90.66	±	10.85	96.86	±	5.14	87.99	±	3.58
	2	20	81.89	±	8.24	91.76	±	3.71	86.83	±	4.77	79.1	±	10.47	95.12	±	5.21	91.23	±	5.7	97.91	±	1.84	90.84	±	3.76
	3	20	73.58	±	27.59	91.89	±	4.53	82.74	±	12.54	67.5	±	17.57	91.71	±	5.63	87.11	±	11.05	97.5	±	3.04	85.95	±	7.11
	1	50	80.63	±	7.33	94.19	±	1.48	87.41	±	3.27	80.68	±	9.94	95.03	±	3.76	90.56	±	4.89	98.53	±	0.73	91.2	±	3.49
	2	50	70	±	22.21	92.97	±	1.69	81.49	±	10.85	75.07	±	17.92	95.32	±	2.73	92.24	±	7.43	98.39	±	1.43	90.26	±	4.24
	3	50	84.84	±	8.04	92.79	±	2.11	88.82	±	4.21	76.91	±	25.38	95.38	±	3.82	94.72	±	4.78	98.2	±	1.26	91.3	±	5.38

Sensitivity and Specificity of set with all PCA features for HS vs PwPD classification and for PwPD staging classification.

By analyzing all the possible combinations, we found that one combination of two features was the minimum set of gait parameters able to distinguish PwPD from controls (knee RoM, trunk rotation RoM) and whose performance (Fig 2A) did not significantly differ (p>0.05) from that of all PCA features.

The results of the independent sample t–test and ROC curve analysis confirmed that the combination of knee and trunk rotation RoM values could significantly differentiate between PwPD and HS (t-statistic = −5.34, p<0.00) and to have good discriminative ability (AUC = 0.77). The numerical sum of knee and trunk rotation RoMs ≤ 66.23 was able to identify PwPD from HS with a 75% probability (Table 3).

**Table 3:** Ability to discriminate between PwPD and HS and between disability levels.

	HS vs PwPD	HY 2 vs 1		HY 3 vs1		HY 4 vs 1		HY 4 vs 3	
	SET	SET1	SET2	SET1	SET2	SET1	SET2	SET1	SET2
AUC	0.77	n.a.	0.73	n.a.	0.76	0.88	0.88	0.77	0.78
OCP	≤ 66.23	n.a.	≤ 105.42	n.a.	≤ 103.91	≤111.71	≤98.09	≤99.28	≤81.35
Se (%)	72.37	n.a.	76.47	n.a.	81.48	91.67	75	66.67	58.35
Sp (%)	73.13	n.a.	60	n.a.	70	80	92	88.89	96.30
LR+	2.69	n.a.	1.91	n.a.	2.72	4.58	3.66	6	15.75
LR-	0.38	n.a.	0.39	n.a.	0.26	0.10	0.11	0.37	0.43
+PTP (%)	75	n.a.	62	n.a.	79	73	69	73	88
-PTP (%)	30	n.a.	25	n.a.	26	6	6	14	16

HS = Healthy Subjects; PwPD = people with Parkinson’s Disease; HY = Hoehn & Yahr disability stage; SET = Knee RoM+Trunk rotation RoM; SET1 = combination of gait speed, hip, knee and ankle Roms values; SET2 = combination of gait speed, hip, knee and trunk rotation RoMs values; AUC = area under the curve; OCP = optimal cutoff point; Se = sensitivity; Sp = specificity; LR+ = positive likelihood ratio; LR- = negative likelihood ratio; +PTP = positive post-test probability: the probability to identify a true positive at OCP; -PTP = negative post-test probability: the probability to identify a false negative at OCP.

### 3.3 PwPD staging classification

The ANNs analysis revealed a mean performance range of 66.16–77.2% (Fig 2B), mean sensibility range of 66–77% (Table 2) and mean specificity range of 85–91% (Table 2) for all features detected by PCA for each number of neurons and HLs.

The number of neurons had no effect on the performance (F_1_ = 2.82, p = 0.099), sensitivity (F_1_ = 0.41, p = 0.522), and specificity (F_1_ = 0.59, p = 0.448) in two-way ANOVA. Furthermore, the HL had no effect on the performances (F_2_ = 0.66, p = 0.522), sensitivity (F_2_ = 0.7, p = 0.499), and specificity (F_2_ = 0.29, p = 0.749) in two-way ANOVA.

By analyzing all the possible combinations, we found that two combinations of four features (walking speed and hip, knee, and ankle RoMs; walking speed and hip, knee, and trunk rotation RoMs) were the minimum set of gait parameters able to distinguish H-Y stage gait patterns from one another and whose performances (Fig 2B) did not differ significantly (p>0.05) from that of all PCA features. All combinations of two or three features showed a significant difference (p<0.05) from that of all PCA features.

The numerical sums of speed, hip RoM, knee RoM, and ankle RoM (SET1) and speed, hip RoM, knee RoM, and trunk rotation RoM (SET2) were able to differentiate PwPD according to H-Y stage (F = 7.59, p<0.00 and F = 9.27, p<0.00, respectively). Post-hoc analysis revealed that SET1 was significantly different between H-Y stages 1 and 4 (p<0.00) and stages 3 and 4 (p = 0.02), while SET2 was able to differentiate between H-Y stages 1 and 2 (p = 0.03), 1 and 3 (p = 0.03), 1 and 4 (p<0.00), and 3 and 4 (p = 0.03). The AUCs, OCPs, sensitivity, specificity, LRs, and post-test probabilities of each set to discriminate PwPD across the H-Y stages are summarized in Table 3.

Briefly, PwPD at H-Y stage 1 were identified by cumulative SET1 values ≥ 111.71 and SET2 values ≥ 105.42, PwPD at H-Y stage 2 by cumulative SET2 values ≥ 103.91 and ≤ 105.42, PwPD at H-Y stage 3 by SET2 values ≥ 81.35 and ≤ 103.91, and PwPD at H-Y stage 4 by SET1 values ≤ 99.28 and SET2 values ≤ 81.35.

## 4. Discussion

Our study was conducted to address the enhancement of the diagnosis and staging of the Parkinsonian gait using an automated machine-learning technique (i.e., ANNs). We found that the accuracy of ANNs obtained with all PCA features did not differ from that obtained with the combination of two features (Fig 2, Table 2); namely, knee and trunk rotation RoMs, suggesting the consideration of only a minimum set of two features to distinguish PwPD from HCs. In the confirmatory analysis, the combination of both knee and trunk rotation RoMs, as the numerical sum of knee and trunk rotation angles, showed a good ability to discriminate PwPD from HCs, with a cumulative threshold value of ≤ 66.23° (Table 3). Knee and trunk rotation RoM abnormalities characterize the gait pattern of PwPD, as found in the current study (Table 1) and in previous studies [53–58], which also revealed a series of other kinematic gait abnormalities. Our findings indicate an ANN algorithm resulted in a drastic reduction in the amount of redundant information, allowing a focus on a few meaningful features that could be used to diagnose and monitor gait function in PwPD. Both the knee and trunk rotation RoMs showed highly discriminatory features characterizing Parkinsonian gait. With regard to the classifier knee joint RoM, our findings are in line with that in study by Caramia et al. [28] using an inertial measurement units-based classification revealed that the knee joint played a major role among the lower limb joints in the assessment of gait in PwPD. PwPD often show lower limb joint rigidity and bradykinesia [59, 60], deficits in knee joint muscle strength and endurance [61, 62], abnormal knee-bent posture [63, 64], and reduced knee motion during walking [65]. Consequently, knee motor control can be impaired in its role of producing an adequate extensor moment [66] and absorbing foot-ground impact [67] in the loading response, recovering and storing energy in the mid-stance [68], and allowing limb progression in the swing phase.

The flexed trunk posture has been reported as a classifier of trunk rotation in PwPD [61–71]. Although the use of the terms camptocormia and Pisa syndrome are restricted to the patients with extreme trunk flexion (>60°) or lateral bending (>10°–15°) [72], who were not included in our study, a certain degree of trunk posture abnormalities is present in most PwPD [73]. In recent decades, a series of quantitative motion analysis studies have also revealed restrained movement of the trunk during several locomotion tasks [74, 75]. Reduced trunk motion combined with postural abnormalities can greatly alter the role of the spine in balance maintenance [6, 68, 75–78], ultimately predisposing patients to falls [71]. Notably, a recent study showed that trunk rotation is a predictor of gait recovery after rehabilitation [57], suggesting that rehabilitation should focus on recovering trunk control [79, 80] to improve both gait and balance [56–58]. Altogether, these findings underline the importance of considering trunk kinematic abnormalities as an integral part of the gait deficit in PwPD.

When classifying the gait deficit of PwPD according to the H-Y staging system, we found that two combinations of four features (SET1: walking speed and hip, knee, and ankle RoMs; SET2: walking speed and hip, knee, and trunk rotation RoMs, respectively,) were the minimum set of gait parameters that were needed to distinguish the gait patterns of PwPD at different H-Y stages (Fig 2, Table 2). Their performance did not significantly differ from that of all PCA features (Fig 2, Table 2). These findings emphasize the usefulness of reduced lower limb joint RoMs as the main classifiers in addition to trunk motion and slow speed, all of which represent sensitive diagnostic features of gait function decline in PwPD. Since our algorithm included 18 gait kinematic variable classifiers, the possibility of focusing on two sets of few gait variables (Fig 2, Table 3) drastically simplifies the diagnosis of gait deficit staging and, thus, might assist physicians in monitoring gait function in PwPD.

Our results should be viewed considering the study limitations and in comparison, with three recent studies on gait classification applying machine-learning techniques in PwPD^28,32,35^. Patients undergoing clinical gait analysis often walk slower than healthy people. Since many gait variables are speed-dependent [79–85], thus, in our study, the control and patient groups were speed-matched to avoid bias due to gait speed. This procedure allowed us to focus on the most discriminative parameters, irrespective of the gait speed. Previous studies on gait deficit classification [28, 31, 34] did not control for gait speed when comparing patients with healthy subjects, and thus, may have given importance to some speed-related classifiers. However, when classifying the gait deficit of PwPD according to the H-Y staging system, we could not control for gait speed. One could argue that joint RoMs might be influenced by the gait speed itself more than the disease severity. However, a robust body of evidence has shown a worsening of lower limb rigidity, bradykinesia [86], and joint RoMs [87] in the advanced stages of the disease, suggesting that reduced gait speed is a consequence of limb rigidity/bradykinesia and not vice versa.

The present results should inspire future research to test the utility of gait classifiers for the differential diagnosis of different neurological gait disorders. Furthermore, longitudinal studies are needed to verify whether the set of gait classifiers represent sensitive tools to monitor the effect of pharmacological and rehabilitation treatments that can modify disease progression.

### 4.1. Limitations

The lack of a comparison of our results with gold standard clinical measures for gait assessment in PwPD as well as with other linear or non-linear statistical models could represent a limitation of this study. Notwithstanding, the probabilities to discriminate PwPD from healthy subjects, and PwPD across the disease progression stages, through the gait parameters reported in our model, is similar to that reported in other studies on PwPD and other neurological disorders [43, 88, 89]. However, there is significant room for improving the accuracy of our model. For instance, other biomarkers of gait impairment, (i.e. arms oscillation, gait variability, kinetic and muscle activation variables) or other dimensions of disability (i.e. cognitive impairment, self-reported quality of life), not included in our analysis, could enhance the accuracy in identifying disability status through gait analysis.
